# Mechanisms and signaling pathways of tyrosine kinase inhibitor resistance in chronic myeloid leukemia: A comprehensive review

**DOI:** 10.1016/j.lrr.2025.100533

**Published:** 2025-08-05

**Authors:** Meriem Lahmouad, Zahrae Rachid, Rawane Bellemrrabet, Jihane Zerrouk, Khan Wen Goh, Abdelhakim Bouyahya, Youssef Aboussalah

**Affiliations:** a Biology and Health Laboratory, Department of Life Sciences, Faculty of sciences, University Ibn Tofail, B.P. 133, Kenitra 14000, Morocco; b Laboratory of Health Sciences and Technologies, Higher Institute of Health Sciences, Hassan First University of Settat, Settat 26000, Morocco; c Flow Cytometry Laboratory, National Institute of Hygiene, Rabat, Morocco; d Faculty of Data Science and Information Technology, INTI International University, Nilai, Malaysia; e Faculty of Mathematics and Natural Sciences, Universitas Negeri Padang, Padang, Indonesia; f Laboratory of Human Pathologies Biology, Department of Biology, Faculty of Sciences, Mohammed V University in Rabat, 10106, Morocco

**Keywords:** Chronic myeloid leukemia, Tyrosine kinase inhibitors, TKI resistance, BCR::ABL1 Mutations, Signaling pathways

## Abstract

Chronic Myeloid Leukemia (CML) is characterized by aberrant BCR::ABL1 tyrosine kinase activity in hematopoietic stem cells. Although tyrosine kinase inhibitors (TKIs) have revolutionized CML treatment, resistance remains a major clinical challenge. This review provides a comprehensive overview of CML, including its epidemiology, pathophysiology, diagnosis, and treatment, as outlined in the latest WHO consensus classification. Current treatment paradigms and the prospects for treatment-free remission (TFR) are also explored. The primary focus is on elucidating the molecular mechanisms of TKI resistance, emphasizing both well-known pathways such as PI3K/AKT, MAPK, JAK/STAT, and alternative pathways including SRC/AKT. This review stands out by integrating recent discoveries regarding genetic mutations within the BCR::ABL1 gene, alongside other molecular alterations contributing to resistance. By synthesizing this knowledge, it aims to guide clinical practitioners, investigators, and translational researchers in developing innovative strategies to overcome resistance and improve patient outcomes in CML.

## Highlights

• Overview of CML: epidemiology, pathophysiology, diagnosis, and WHO classification updates.
• Focus on TKI resistance: PI3K/AKT, MAPK, JAK/STAT, SRC/AKT pathways.
• Insights into genetic mutations driving resistance, advancing clinical strategies for CML.

## 1 Introduction

CML is a myeloproliferative neoplasm characterized by the uncontrolled proliferation of granulocytes, a subtype of white blood cells. This condition arises from a single hematopoietic stem cell in the bone marrow and is marked by an overproduction of these cells, disrupting the normal balance of blood cell types [1]. The defining feature of CML is the Philadelphia chromosome, a genetic anomaly resulting from a translocation between chromosomes 9 and 22, which leads to the formation of the BCR::ABL fusion gene. This gene encodes an oncoprotein with persistent tyrosine kinase activity, which drives the uncontrolled proliferation and survival of myeloid cells. Consequently, targeting the BCR::ABL oncoprotein is crucial for the diagnosis, monitoring, and treatment of CML [2].

The advent of TKIs such as imatinib has revolutionized the management of CML, significantly enhancing patient outcomes and extending life expectancy. Despite these advancements, TKI resistance poses a substantial challenge, impacting approximately 20–30 % of patients receiving first-line therapies. Resistance mechanisms can be broadly categorized into BCR::ABL1-dependent pathways, including mutations within the BCR::ABL1 kinase domain, and BCR::ABL1-independent pathways, which involve alternative signaling cascades. Recent studies have identified critical pathways such as PI3K/AKT, MAPK, and JAK/STAT as key players in the development of resistance, alongside emerging pathways like SRC/AKT, which warrant further exploration for therapeutic targeting [3].

In addition to the biological complexities of CML, the economic implications of managing resistant cases are significant. The treatment of resistant CML often necessitates the use of second and third-generation TKIs, which can escalate annual treatment costs by 50–100 % compared to first-line therapies. For instance, a recent analysis highlighted the financial burden associated with TKI treatment failures, revealing that healthcare costs increase substantially with each subsequent line of therapy, driven primarily by hospitalizations and outpatient services [4,5]. This underscores the urgent need for innovative strategies to mitigate resistance, which, while potentially increasing initial treatment costs, may ultimately reduce long-term healthcare expenditures by preventing disease progression and minimizing the need for salvage therapies.

CML has an incidence of approximately 1–2 cases per 100,000 individuals annually, with a male-to-female ratio of 1.4:1. The median age at diagnosis in Western populations is around 65 years. In contrast, emerging countries report a significantly lower median age at diagnosis, often ranging from 10 to 20 years younger than in Western populations, which may contribute to a more aggressive disease course in younger patients. Notably, in emerging countries, the median age at diagnosis is significantly lower, often ranging from 10 to 20 years younger than in Western populations, which may contribute to a more aggressive disease course in younger patients [6].

The diagnosis of CML is frequently incidental, identified through routine blood tests showing leukocytosis and basophilia. When symptomatic, patients commonly present with fatigue, weight loss, night sweats, and splenomegaly. The definitive diagnosis relies on detecting the BCR::ABL1 fusion gene using cytogenetic and molecular techniques [7] and its staging follows the revised 2022 WHO classification, which simplifies the disease into two phases: chronic phase (CP) and blast phase (BP), with the previously recognized accelerated phase now considered "high-risk CP" [8]. Prognostic scores such as Sokal, Hasford, and EUTOS are used to stratify patients based on clinical and laboratory parameters. It is noteworthy to mention that high-risk additional chromosomal abnormalities (ACAs) also contribute to disease progression and treatment resistance [8].

**Fig. 1:**
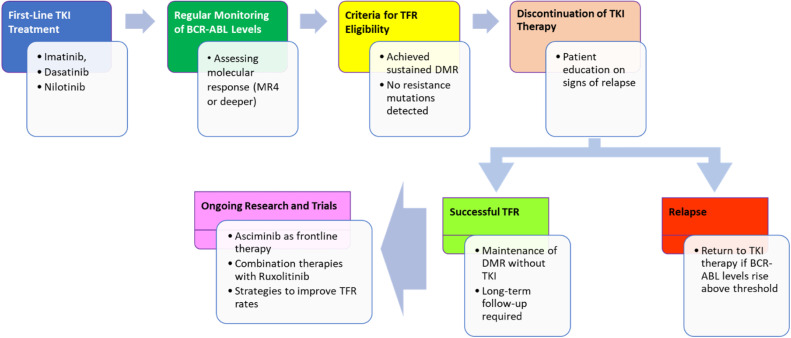
Therapeutic pathway to achieving therapy-free remission in chronic myeloid leukemia.

Building on these diagnostic foundations, TKIs have dramatically improved the 10 year survival rate for CML patients to approximately 80–90 %. First-line therapy includes imatinib, while second- and third-generation TKIs (e.g., dasatinib, nilotinib, bosutinib, ponatinib) are used in cases of resistance or intolerance [8]. Recently, asciminib, an allosteric BCR::ABL1 inhibitor, has emerged as a promising therapeutic option. While treatment-free remission (TFR) is achievable in select patients, resistance to TKIs often due to mutations like T315I or activation of alternative signaling pathways remains a major obstacle (Fig. 1).

This review aims to delve into the molecular mechanisms underlying TKI resistance in CML, focusing on established signaling pathways and the potential for novel therapeutic strategies that could enhance patient prognosis. By synthesizing recent research findings, we hope to illuminate the complexities of CML resistance and may contribute to developing more effective treatment paradigms to improve the quality of life for patients afflicted by this challenging disease. This review adopts a systematic approach, prioritizing the synthesis of the most relevant and up-to-date literature on TKI resistance in CML. We partially adapted the PRISMA guidelines to ensure transparency and rigor in the methodology [9]. A comprehensive bibliometric analysis was conducted using the PubMed database, employing the following keywords; CML, TKI resistance, signaling pathways, BCR::ABL mutations, and alternative therapies. The inclusion criteria focused on studies published primarily after 2022, following the release of the updated WHO classification of myeloid neoplasms. These studies emphasized human clinical trials, molecular investigations, and reviews that specifically explored the mechanisms of TKI resistance and potential therapeutic interventions. Articles that focused on unrelated hematologic malignancies or lacked specific insights into signaling pathways and resistance mechanisms in CML were excluded. By employing this methodology, we ensured the integration of the most pertinent and cutting-edge research, providing a robust and comprehensive foundation for this review (Table 1).

**Table 1:** Clinical studies evaluating various TKIs in patients with relapsed or refractory chronic myeloid leukemia.

Reference	Phase	Study Schema	Population Size	Overall Response Rate (ORR)	Time to Progression/Progression-Free Survival (PFS)	Notes
[10]	I/II	Dose-escalation study of ponatinib	449	70 %	Median PFS: 22 months	Patients with relapsed/refractory CML; showed durable responses
[11]	II	Dasatinib for imatinib-resistant/intolerant CML	387	63 %	2-year PFS: 63 %	Patients with resistance or intolerance to imatinib; demonstrated efficacy and safety
[12]	II	Nilotinib in patients with resistance to other TKIs	321	59 %	24-month PFS: 59 %	Patients resistant or intolerant to imatinib; showed effective cytogenetic and molecular responses
[13]	III	Bosutinib vs. imatinib in previously treated CML	546	Bosutinib: 73 %, Imatinib: 60 %	2-year PFS: Bosutinib 79 %, Imatinib 71 %	Patients who failed prior imatinib therapy; Bosutinib showed superior efficacy and faster cytogenetic response
[7]	III	Ponatinib vs. imatinib in resistant/intolerant CML	270	Ponatinib: 69 %, Imatinib: 54 %	2-year PFS: Ponatinib 83 %, Imatinib 74 %	Patients resistant or intolerant to dasatinib or nilotinib, or with T315I; Ponatinib showed superior efficacy but with increased risk of arterial occlusive events
[14]	III	Asciminib vs. bosutinib in TKI-resistant CML	233	Asciminib: 77 %, Bosutinib: 52 %	2-year PFS: Asciminib 86 %, Bosutinib 68 %	Patients resistant or intolerant to ≥2 prior TKIs; Asciminib demonstrated superior MMR and lower rates of adverse events
[15]	III	Efficacy of second-generation TKIs in CML	450	75 %	3-year PFS: 75 %	This article discusses the efficacy of second-generation TKIs in CML, so individual trial details are unavailable. This value reflects overall efficacy across multiple trials.
[16]	II	Evaluating the efficacy of Olverembatinib in CML	300	82 %	12-months PFS: 70 %	This study was on patients with CML with T315I mutation who had failed prior TKI therapies. The ORR and PFS are specific to this population.
[14]	III	Asciminib vs. Bosutinib	233	Asciminib MMR 25.5 % vs Bosutinib 13.2 %	N/A	CML-CP patients resistant or intolerant to ≥2 prior TKIs; Asciminib demonstrated superior MMR rate at 24 weeks compared to bosutinib
[17]	II	Ponatinib (different starting doses)	283	Varies by Dose	N/A	CML-CP or AP patients resistant or intolerant to prior TKIs; Dose-dependent efficacy and toxicity (OPTIC Trial).
[18]	II	Ponatinib	449	70 %	N/A	CML patients resistant or intolerant to dasatinib or nilotinib, or with the T315I mutation; Ponatinib demonstrated significant efficacy in heavily pretreated CML patients (PACE trial).
[19]	III	Nilotinib vs. Imatinib	846	Nilotinib Superior	N/A	Newly diagnosed CML-CP patients; Nilotinib showed superior MMR rates compared to imatinib.
[20]	III	Dasatinib vs. Imatinib	519	Dasatinib Superior	N/A	Newly diagnosed CML-CP patients; Dasatinib demonstrated higher CCyR rates compared to imatinib.
[21]	II	Ruxolitinib + TKI (various)	45	64 % (MMR)	N/A	CML patients with suboptimal response to TKI therapy; The combination of ruxolitinib and TKI showed encouraging activity - SPIRIT trial
[22]	II	Venetoclax + TKI	25	76 % (MMR)	N/A	CML patients with relapse after TKI discontinuation; Venetoclax with TKI achieved deep molecular response.
[14]	Phase III	Asciminib vs. Bosutinib	233	Asciminib MMR 25.5 % vs Bosutinib 13.2 %	N/A	CML-CP patients resistant or intolerant to ≥2 prior TKIs; Asciminib demonstrated superior MMR rate at 24 weeks compared to bosutinib
[18]	Phase II	Ponatinib (different starting doses)	283	Varies by Dose	N/A	CML-CP or AP patients resistant or intolerant to prior TKIs; Dose-dependent efficacy and toxicity (OPTIC Trial).
[23]	Phase II	Ponatinib	449	70 %	N/A	CML patients resistant or intolerant to dasatinib or nilotinib, or with the T315I mutation; Ponatinib demonstrated significant efficacy in heavily pretreated CML patients (PACE trial).
[24]	Phase III	Nilotinib vs. Imatinib	846	Nilotinib Superior	N/A	Newly diagnosed CML-CP patients; Nilotinib showed superior MMR rates compared to imatinib.
[25]	Phase III	Dasatinib vs. Imatinib	519	Dasatinib Superior	N/A	Newly diagnosed CML-CP patients; Dasatinib demonstrated higher CCyR rates compared to imatinib.
[26]	Phase II	Ruxolitinib + TKI (various)	45	64 % (MMR)	N/A	CML patients with suboptimal response to TKI therapy; The combination of ruxolitinib and TKI showed encouraging activity - SPIRIT trial
[27]	Phase II	Venetoclax + TKI	25	76 % (MMR)	N/A	CML patients with relapse after TKI discontinuation; Venetoclax with TKI achieved deep molecular response.

## 2 Mechanisms of resistance in chronic myeloid leukemia

**Fig. 2:**
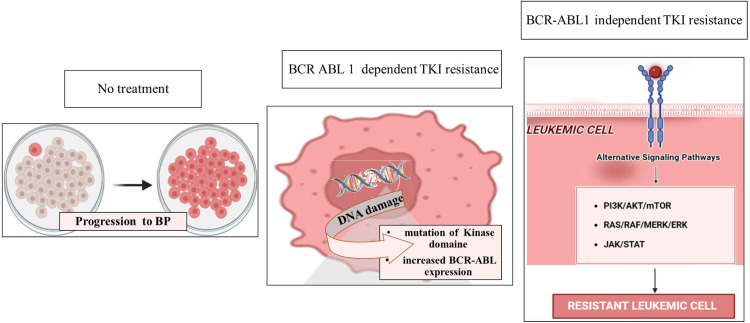
Pathways of tyrosine kinase inhibitor resistance in chronic myeloid leukemia. Modified from [21] using Biorander software.

Despite the remarkable success of TKIs in targeting BCR-ABL1 and transforming the treatment of CML, resistance remains a significant clinical challenge. Resistance can be classified into two main categories: BCR::ABL1-dependent and BCR::ABL1-independent pathways. In addition to mutations, increased BCR::ABL1 expression, often driven by BCR::ABL1 gene amplification, can contribute to resistance. Higher levels of the BCR::ABL1 protein can overwhelm the inhibitory capacity of TKIs, leading to reduced treatment efficacy. This mechanism should be considered, especially in patients exhibiting increasing BCR::ABL1 transcript levels despite TKI therapy [28] (Fig. 2).

Main pathways of tyrosine kinase inhibitor (TKI) resistance in chronic myeloid leukemia (CML), distinguishing between BCR::ABL1-dependent and independent mechanisms. Resistance in the chronic phase to blast phase progression is driven by kinase domain mutations and BCR::ABL1 overexpression (dependent) or activation of alternative signaling pathways such as PI3K/AKT/mTOR, RAS/RAF/MEK/ERK, and JAK/STAT (independent). *BCR::ABL1,* Breakpoint Cluster Region-Abelson 1; *TKI,* Tyrosine Kinase Inhibitor; *PI3K,* Phosphatidylinositol 3-Kinase; *AKT,* Protein Kinase B; *mTOR,* Mechanistic Target of Rapamycin; *RAS,* Rat Sarcoma Virus Gene; *RAF,* Rapidly Accelerated Fibrosarcoma; MEK, Mitogen-Activated Protein Kinase; ERK, Extracellular Signal-Regulated Kinase; *JAK,* Janus Kinase; *STAT,* Signal Transducer and Activator of Transcription; *BP,* Blast Phase.

### 2.1 BCR::ABL1-dependent resistance

BCR::ABL1-dependent resistance primarily arises from mutations within the kinase domain of the BCR::ABL1 protein. These mutations directly impact the TKI binding site, altering the affinity of TKIs, rendering them less effective or completely ineffective [29]. Over 90 distinct mutations have been identified across the BCR::ABL1 kinase domain, each with varying degrees of TKI sensitivity. The T315I mutation is particularly notorious due to its unique resistance profile, conferring near-complete resistance to most TKIs, with the exception of ponatinib and asciminib. This mutation arises from a threonine to isoleucine substitution at position 315, creating steric hindrance that prevents the binding of most TKIs [30]. Other clinically significant mutations include Y253H, E255K, F317L, and G250E, which can compromise the efficacy of second-generation TKIs like dasatinib and nilotinib. The Y253H mutation, for example, introduces a bulky tyrosine residue that interferes with dasatinib binding (Huang, Xiao et al. 2024). Compound mutations, where two or more mutations exist in the same BCR::ABL1 allele, are also increasingly recognized as mechanisms of resistance, often leading to more pronounced TKI insensitivity.

The mechanisms by which these mutations induce resistance vary but often involve steric hindrance, conformational changes, or alterations in the electrostatic interactions between the TKI and the BCR::ABL1 protein [31]. As mentioned earlier, the T315I mutation replaces a threonine residue with isoleucine at position 315, disrupting the binding of most TKIs due to steric hindrance [30]. In contrast, the Y253H mutation introduces a bulky tyrosine residue, which can disrupt the binding of dasatinib and nilotinib. E255K alters the electrostatic environment, reducing the binding affinity of imatinib and dasatinib [32].

Understanding the specific mutation profile of a patient is crucial for selecting the most appropriate TKI therapy and predicting treatment outcomes. Next-generation sequencing (NGS) technologies have enabled more rapid and comprehensive mutation analysis, allowing for personalized treatment strategies [32]. For example, patients with the T315I mutation are typically treated with ponatinib or asciminib, while those with mutations sensitive to second-generation TKIs, such as Y253H or E255K, may benefit from dasatinib or nilotinib. Patients with compound mutations often require more aggressive treatment strategies, including allogeneic stem cell transplantation or experimental therapies [32].

### 2.2 BCR::ABL1-independent resistance

BCR::ABL1-independent resistance involves alternative survival pathways that enable leukemic cells to evade TKI treatment. While these pathways, including PI3K/AKT/mTOR, RAS/RAF/MEK/ERK, and JAK/STAT, are indeed activated in response to various stimuli during hematopoiesis and play fundamental roles in development and differentiation, their activation in the context of TKI resistance is particularly relevant when considering mutations or upregulation of their components [33]. It is essential to recognize that the activation of these pathways is not solely due to TKI inhibition; rather, it reflects a complex interplay of cellular signaling that allows leukemic cells to survive despite BCR::ABL1 inhibition. Recent studies have highlighted the importance of alterations in phosphatase gene expression and function in promoting TKI insensitivity in CML cells. Phosphatases such as PP2A, SHP1, and PTPN22 have been implicated in modulating BCR::ABL signaling and influencing TKI response. For instance, decreased expression of PP2A has been associated with TKI resistance, while restoration of its activity can enhance TKI sensitivity [34].

The clinical implications of these alternative pathways are significant. For instance, patients exhibiting resistance due to PI3K/AKT/mTOR pathway activation may benefit from a combination of TKIs with mTOR inhibitors, specifically targeting this survival pathway. Similarly, inhibitors that target the JAK/STAT pathway could offer therapeutic options for patients resistant to standard TKI therapy [35]. Furthermore, deubiquitinating enzymes have been implicated in resistance mechanisms by regulating protein stability and degradation pathways that affect BCR::ABL1 signaling [36] (Table 2). In addition to these resistance mechanisms, pharmacogenetic variants in cytochrome P450 enzymes (CYP3A4 and CYP3A5) can influence TKI metabolism and efficacy based on the patient's liver function. This highlights the stochastic fluctuations between drug efficacy and toxicity, which depend on the liver's capacity to metabolize TKIs according to the patient's genomic profile [36]. Therefore, while discussing resistance mechanisms, it is crucial to also consider pharmacokinetic factors that may affect treatment outcomes. Furthermore, understanding the mechanisms of resistance has led to the development of second- and third-line therapies that are more effective against resistant CML cases. For patients who develop resistance to imatinib, second-generation TKIs such as dasatinib, nilotinib, and bosutinib provide alternative options, each with distinct efficacy profiles and side effect considerations. For example, dasatinib has demonstrated efficacy in patients with mutations in the P-loop region, while nilotinib is often preferred in patients with cardiovascular risk factors, given its more favorable side effect profile in this regard [37,38]. Ponatinib, a third-generation TKI, remains the gold standard for patients with the T315I mutation, but its use must be balanced against the risk of arterial occlusive events [39]. Asciminib, a novel allosteric inhibitor of BCR::ABL1 that targets the myristoyl pocket rather than the ATP-binding site, offers a new mechanism of action that may overcome resistance in patients with multiple mutations [37]. Eventhough, recent findings indicate that resistance mutations can still emerge within the myristoyl site, even when Asciminib is used as a first-line therapy [40]. For instance, new myristoyl-pocket mutations were detected in 2 of 20 patients who had disease progression during asciminib treatment and in 2 of 66 patients without evidence of disease progression who had received asciminib for at least 12 months. One patient with chronic-phase CML and a baseline E255K mutation eventually developed a myristoyl-pocket G463S mutation after 50 weeks of treatment, highlighting the potential for resistance development even during extended therapy.

**Table 2:** Signaling pathways that play a role in the acquirement of TKI-resistance.

Signaling Pathway/Mechanism	Study Design	Sample Size	Outcomes	Reference
BCR::ABL Mutations	Clinical	200	55 % of imatinib-resistant patients had mutations in the BCR::ABL kinase domain; significant impact on treatment response. Recent studies indicate that mutations are present in 40 % to 60 % of cases of secondary resistance.	[10]
SRC Family Kinases (SFKs) Activation	Preclinical	*In vitro*	Increased SFK activity associated with resistance; dasatinib effectively inhibited SFK signaling, restoring sensitivity.	[41]
Phosphoinositide 3-Kinase (PI3K)/AKT Pathway	Clinical	100	Activation of the PI3K/AKT pathway correlated with poor response to imatinib; targeting this pathway improved outcomes in resistant cases.	[42]
Mitogen-Activated Protein Kinase (MAPK) Pathway	Preclinical	*In vitro*	MAPK pathway activation observed in imatinib-resistant cells; inhibition of this pathway led to reduced cell proliferation.	[43]
Increased BCR::ABL Expression	Clinical	150	Higher BCR::ABL expression levels linked to resistance; necessitated the use of second-line therapies like ponatinib.	[2]
Janus Kinase (JAK) Pathway Activation	Preclinical	*In vitro*	JAK pathway activation contributed to resistance mechanisms; targeting JAK showed potential in overcoming resistance.	[44]
Deubiquitinating Enzymes	Preclinical	*In vitro*	Deubiquitinating enzymes are identified as potential biomarkers of TKI resistance, regulating key signaling pathways that contribute to cell survival under therapeutic pressure.	[45]
Cytochrome P450 Variants	Clinical	Varied	Pharmacogenetic variants in cytochrome P450 enzymes (CYP3A4 and CYP3A5) can alter TKI pharmacokinetics, influencing patient responses to treatment.	[33]

In cases where resistance persists despite multiple lines of TKI therapy, allogeneic stem cell transplantation (AlloSCT) remains the only curative option. However, this approach is limited by donor availability and high associated risks, such as graft-versus-host disease [33]. Therefore, the focus of ongoing research is to improve the effectiveness of TKIs and identify combination strategies that target both BCR::ABL1-dependent and independent mechanisms.

### 2.3 Other mechanisms of resistance in CML

#### 2.3.1 Leukemia stem cell persistence

Leukemia stem cells (LSCs) play a crucial role in both primary and secondary resistance, as they often survive TKI treatment and contribute to disease relapse. These cells are maintained through signaling pathways like Hedgehog and Notch, which regulate their self-renewal and survival [46]. During blast-stage CML, increased BCR::ABL1 expression leads to elevated levels of nuclear and cytoplasmic β-catenin, a key regulator of leukemic stem cell self-renewal and survival [47]. Aberrant β-catenin activation has been closely linked to disease progression and TKI resistance, contributing to the persistence of leukemic clones despite therapy [48]. Recent evidence suggests that β-catenin plays a central role in maintaining the leukemic stem cell population, similar to the Hedgehog and Notch pathways[49]. Consequently, therapeutic strategies that combine TKIs with β-catenin inhibitors may offer a promising approach to eradicating residual leukemic stem cells and overcoming resistance.

Targeting these pathways could offer a strategy to eradicate LSCs, thereby reducing the risk of relapse and improving long-term outcomes. Clinical trials investigating Hedgehog pathway inhibitors and other LSC-targeted therapies are ongoing, and their integration into treatment regimens may be pivotal in overcoming resistance [50].

#### 2.3.2 Drug transporters

The effectiveness of TKIs is influenced by drug transporters that regulate drug influx and efflux. OCT1, a solute carrier (SLC) transporter encoded by the SLC22A1 gene, is a key mediator of TKI uptake. Higher OCT1 expression is associated with major molecular responses, while low expression is linked to multidrug resistance and suboptimal responses in CML [51]. Other transporters, such as OCTN2, OATPs, and MATE1, also contribute to TKI transport [52]. In cases of imatinib resistance, a decrease in OCT1 and OCTN2 expression has been observed, highlighting the involvement of multiple influx transporters in the resistance process [51]. Conversely, ATP-binding cassette (ABC) transporters, such as P-glycoprotein (P-gp) and breast cancer resistance protein (BCRP), mediate the extrusion of metabolites and chemotherapeutic agents, affecting intracellular drug concentrations and treatment efficacy (Pote and Gacche 2023). Overexpression of these transporters is associated with poor outcomes and resistance to TKIs in CML [53]. Understanding the interplay between drug transporters, genetic variants, and TKI uptake is crucial in addressing resistance mechanisms in CML. Lower drug uptake or increased drug extrusion can create an environment conducive to the development of additional resistance mechanisms, such as BCR::ABL1 mutations, emphasizing the importance of personalized treatment strategies targeting drug transporters to optimize therapeutic outcomes in CML.

#### 2.3.3 DNA damage repair and genomic instability

Deregulation of DNA damage response (DDR) pathways leads to genomic instability, facilitating resistance development and disease progression. The BCR::ABL1 oncoprotein induces genomic instability by generating reactive oxygen species (ROS) and activating error-prone DNA repair mechanisms[54]. This results in the accumulation of DNA damage, which can promote the survival of leukemic cells despite TKI treatment. Recent studies have highlighted the role of various DNA damage response (DDR) genes in mediating therapeutic outcomes. For instance, genes involved in double-strand break (DSB) repair mechanisms, such as BRCA1, RAD51, and POLQ, have been associated with poor prognosis in CML patients undergoing TKI treatment [55]. The upregulation of these genes indicates that CML cells may rely on error-prone repair pathways, such as non-homologous end joining (NHEJ), to survive the DNA damage induced by therapy [21]. Additionally, the BCR::ABL fusion protein, which is central to CML pathogenesis, has been shown to mediate genomic instability through the activation of various DNA repair pathways, leading to further mutations and chromosomal aberrations [56]. This genomic instability is exacerbated by oxidative stress and reactive oxygen species (ROS), which are prevalent in CML cells and can lead to additional DNA damage if not properly repaired [38].

Furthermore, altered expression levels of genes such as CDC42BPA are required for TP53-dependent autophagy and AIM2, which play a crucial role in the innate immune response by forming the AIM2 inflammasome to facilitate pyroptosis. They have been implicated in the DNA damage response, suggesting that these alterations may serve as potential biomarkers for predicting treatment outcomes in CML patients [55]. Understanding these altered DNA repair mechanisms is crucial for developing targeted therapies that can effectively address resistance in CML. Overall, the dysregulation of DDR mechanisms directly impacts TKI resistance and CML progression, emphasizing the importance of understanding and targeting these pathways in CML management. On the other hand, recent research has identified altered CD genes and markers (as summarized in Table 3), such as high CD302 expression and the presence of CD34+CD302+ and CD14+CD302+ cells, contributing to TKI’s resistance [57,58]. Furthermore, T cell subpopulations like CD4-FOXP3, CD8-GZMA, and CD8-GNLY, along with IDO1 in myeloid cells, may also further complicate the resistance mechanisms [[59], [60], [61]]. Collectively, these findings underscore the importance of identifying specific genes and markers associated with TKI resistance, which could inform the development of targeted therapies aimed at overcoming treatment failure in CML patients.

**Table 3:** Association of CD genes and markers with imatinib resistance in chronic myeloid leukemia.

CD Gene/Marker	Association with Imatinib Resistance	References
CD302	High expression linked to inferior achievement of deep molecular response (DMR) in imatinib and nilotinib treatment. Patients with high CD302 expression had a DMR achievement rate of 0 % compared to 83 % in those with low expression.	[57]
CD34+CD302+ Cells	Higher presence in patients with poor responses to TKIs, indicating potential contribution to leukemic cell persistence and resistance.	[58]
CD14+CD302+ Cells	Similar to CD34+CD302+, higher levels found in patients with poor responses, suggesting a role in resistance mechanisms.	[58]
CD4-FOXP3 & MKI67	Analyzed in imatinib-resistant gastrointestinal stromal tumors (GISTs), indicating a potential role in resistance.	[61]
CD8-GZMA & MKI67	Found in T cell subpopulations analyzed in imatinib-resistant GISTs, suggesting involvement in resistance pathways.	[61]
CD8-GNLY	Another T cell subpopulation associated with resistance in imatinib-resistant GISTs.	[60]
IDO1_DC (myeloid cells)	More distributed in imatinib-resistant GIST tumor microenvironments compared to sensitive ones, indicating a role in resistance.	[59]

#### 2.3.4 Epigenetic alterations

Epigenetic dysregulation plays a significant role in the pathogenesis and progression of CML. While mutations in epigenetic regulating genes, such as DNMT3A, TET2, EZH2, and ASXL1, are relatively rare in chronic phase CML, they become more prevalent during disease progression[2]. Epigenetic modifications, which involve the addition or removal of small molecules like methyl or acetyl groups on DNA or histones, lead to chromatin remodeling and altered gene expression [62]. It is well established that the hematopoietic differentiation program is influenced by de novo DNA methyltransferases (DNMTs), which establish new methylation patterns during cell differentiation [62]. Maintenance DNMTs are essential for preserving cellular memory by ensuring that established methylation patterns are faithfully copied during DNA replication. This cellular memory is crucial for maintaining the identity and function of hematopoietic cells. TET enzymes also play a key role in the differentiation-memory transition by regulating demethylation profiles. These enzymes convert 5-methylcytosine to 5-hydroxymethylcytosine, facilitating active demethylation processes that are critical for gene expression during differentiation [63]. The balance between DNMTs and TET enzymes is vital for proper hematopoietic cell function and can significantly influence resistance mechanisms in CML. In addition to these epigenetic factors, metabolic and energetic reprogramming are strongly linked to resistance mechanisms. The metabolic state of a cell affects the activity of both TET enzymes and DNMTs, impacting their ability to modify DNA methylation patterns effectively [62]. Alterations in cellular metabolism can lead to changes in the availability of substrates necessary for these enzymatic reactions, thus affecting gene expression profiles associated with drug resistance. DNA hypermethylation, particularly of genes like p15, RASSF1A, TFAP2A, and EBF2, is a common oncogenic process observed in CML, especially in advanced phases of the disease [64]. Histone modifications, such as acetylation and methylation, also play a crucial role in modulating gene expression in CML cells [65]. Furthermore, post-translational processes, especially microRNAs, are essential in epigenetic regulation in CML. MicroRNAs block protein synthesis and promote mRNA degradation, influencing gene expression patterns in CML cells. Distinct microRNA expression profiles have been observed in CML patients compared to healthy individuals, with variations between different disease phases and treatment responses [66] (Table 4). The phenotypic changes and molecular mechanisms associated with TKI resistance in CML highlight the complexity of this disease and the challenges in achieving effective treatment outcomes. Understanding the role of CD expression, DNA damage repair, drug transporters, and epigenetic alterations is crucial for developing targeted strategies to overcome resistance. Continued research in these areas will be essential for advancing CML management and improving patient prognosis.

**Table 4:** Key epigenetic changes associated with TKI resistance in CML.

Epigenetic Change	Mechanism	Impact on TKI Resistance	Ref
**DNA Hypermethylation**	Methylation of tumor suppressor genes (e.g., p15, RASSF1A)	Silencing of genes that regulate cell cycle and apoptosis, promoting survival	[64]
**Histone Modifications**	Acetylation and methylation of histones (e.g., acetylation of histone H3 at K27 activates enhancers; methylation at H3K9 silences tumor suppressor genes)	Altered chromatin structure leading to aberrant gene expression	[65,67]
**MicroRNA Dysregulation**	Changes in microRNA expression profiles(e.g., upregulation of miR-21 inhibits pro-apoptotic factors, enhancing resistance to therapy)	Inhibition of target genes involved in apoptosis and proliferation	[66]
**Mutations in Epigenetic Regulators**	Mutations in genes like DNMT3A, TET2	Disruption of normal epigenetic regulation, contributing to resistance	[62]

## 3 Emerging therapeutic approaches

The landscape of CML treatment continues to evolve, with researchers and clinicians exploring novel strategies to overcome TKI resistance and improve patient outcomes. Recent advancements have opened up promising avenues for therapeutic intervention, offering new hope for patients who do not respond to conventional therapies.

One such advancement is the use of BCL-2 inhibition, which has emerged as a potential game-changer in targeting CML stem cells resistant to TKIs. Venetoclax, a selective BCL-2 inhibitor, has shown promise in preclinical studies and early clinical trials. By targeting the anti-apoptotic protein BCL-2, which is overexpressed in CML stem cells, venetoclax may help eliminate residual disease and reduce the risk of relapse [68]. However, monotherapy approaches have limitations, leading to growing interest in combination therapies that integrate TKIs with immunotherapies.

The rationale behind combining TKIs with immune checkpoint inhibitors is to leverage the immune system’s ability to recognize and eliminate leukemic cells while simultaneously inhibiting BCR::ABL1. Ongoing clinical trials evaluate combinations of TKIs with agents such as anti-PD-1 and anti-CTLA-4 antibodies, aiming to enhance treatment efficacy and counteract resistance mechanisms[69]. In parallel, CAR T-cell therapy, which has demonstrated remarkable success in other hematologic malignancies, is now being explored for TKI-resistant CML [70]. Early-phase trials are assessing CAR T-cells targeting CML-specific antigens, intending to offer a potentially curative option for patients who have exhausted other treatment alternatives.

In addition to immunotherapies, researchers are developing novel BCR::ABL1 inhibitors that provide greater efficacy against resistant mutations. Asciminib, an allosteric inhibitor that binds to a different site on the BCR::ABL1 protein compared to traditional TKIs, has shown promise in clinical trials, particularly for patients harboring the T315I mutation. This targeted approach highlights the ongoing efforts to refine TKI therapy and improve patient outcomes [71].

Beyond direct kinase inhibition, epigenetic modifiers are being investigated as a means to resensitize resistant CML cells to TKIs. Histone deacetylase inhibitors and DNA methyltransferase inhibitors have demonstrated synergistic effects with TKIs in preclinical models, suggesting that modifying gene expression may enhance the efficacy of existing treatments [72,73]. Building on this concept, synthetic lethality is emerging as another promising strategy, in which researchers aim to identify and target genes or pathways that, when inhibited alongside BCR::ABL1, selectively induce leukemic cell death while sparing normal cells.

Alongside these molecularly targeted therapies, increasing attention is being given to the tumor microenvironment and its role in CML progression and drug resistance. Leukemic cells interact with the bone marrow niche in ways that can promote survival and limit TKI efficacy. Strategies aimed at disrupting these protective interactions, such as CXCR4 inhibitors, are being explored to enhance the impact of TKI therapy by weakening the supportive environment that sustains resistant leukemic cells [74].

Together, these emerging approaches reflect the ongoing efforts to address the challenges of TKI resistance and optimize treatment strategies for CML. As research progresses, the future of CML therapy will likely involve personalized treatment regimens that incorporate a combination of these novel interventions, tailored to individual patient characteristics and specific resistance mechanisms.

## 4 Conclusion and future perspectives

The introduction of TKIs has revolutionized the treatment of CML by specifically targeting the BCR::ABL1 oncoprotein. This advancement has improved patient survival rates and reshaped the management of CML. However, despite these significant gains, TKI resistance remains a critical challenge. The underlying mechanisms of resistance are complex, involving mutations in the drug target, variations in drug levels, alterations in leukemic signaling pathways, and changes in the tumor microenvironment and immune function, all of which affect treatment efficacy. While mutations in the BCR::ABL1 protein are a major cause of resistance, BCR::ABL1-independent mechanisms also play an important role, underscoring the need for a deeper understanding of alternative pathways involved in treatment response (Kantarjian, Jabbour, and O'Brien, 2024). Identifying biomarkers that predict patient response to treatment is crucial for optimizing therapy. Some of these biomarkers may also serve as targets for new treatments. Additionally, genetic variations such as single nucleotide variants (SNVs) influence disease risk, prognosis, and treatment outcomes, adding further complexity to CML management [75]. While hematopoietic stem cell transplantation remains the only curative option for CML, its use is limited due to high toxicity and the challenge of finding suitable donors [76].

It is worth mentioning that although CML has traditionally been viewed as a disease affecting older adults, there is an increasing incidence among younger populations, including adolescents and young adults. Recent statistics show that 10–20 % of new CML cases now occur in individuals under 30, compared to the median diagnosis age of around 60 years [77]. This shift necessitates longer treatment durations, increasing the likelihood of TKI resistance and faster progression to the blast phase. Younger patients, facing a longer post-diagnosis life expectancy, are particularly impacted by these challenges. Therefore, it is essential to develop new treatment strategies tailored to the specific needs of younger patients, especially in addressing TKI resistance. On the other hand, mutations in the BCR::ABL1 protein, such as the T315I mutation, are key drivers of TKI resistance (Kaleem, Shahab, and Shamsi, 2024). To overcome this, more flexible treatment approaches are required. These include targeting alternative sites on BCR::ABL1 and developing drugs that bind differently from Imatinib, such as Asciminib, which targets specific pockets of the BCR::ABL1 protein. Also, Combination therapies involving multiple TKIs or pairing TKIs with other drugs targeting different molecular pathways are a promising avenue. Additionally, exploring the metabolic changes that affect TET enzymes and DNMTs, which regulate gene expression in TKI-resistant patients, is necessary. While addressing resistance remains a priority, long-term safety considerations must also be integrated into future therapeutic strategies. Prolonged TKI use has been associated with adverse effects such as cardiovascular complications, metabolic disorders, and organ toxicity, which can significantly impact patients’ quality of life. Balancing treatment efficacy with minimizing toxicity is a crucial challenge, particularly for younger patients who require decades of therapy. Future research should not only focus on enhancing drug potency but also on optimizing treatment schedules, exploring drug discontinuation strategies, and identifying biomarkers that predict both resistance and adverse effects to personalize treatment approaches.

Beyond the biological and clinical challenges of resistance, the economic implications of TKI resistance are also substantial. Resistance often necessitates the use of second- and third-generation inhibitors, which are more costly, and increases the risk of progression to the blast phase. This stage of the disease is associated with a much poorer prognosis and requires aggressive treatments such as chemotherapy and allogeneic stem cell transplantation, further escalating costs. Therefore, developing cost-effective, clinically applicable strategies is essential. A comparative flowchart summarizing drug efficacy, resistance rates, and associated costs could aid clinical decision-making, helping to balance therapeutic benefits with economic considerations.

The growing availability of next-generation TKIs and combination therapies offers potential but also presents challenges due to high costs and varied side-effect profiles. Personalized treatment algorithms, taking into account drug efficacy, cost, and patient-specific risk factors, are essential for optimizing long-term outcomes

Addressing these concerns, along with integrating comprehensive genetic profiling to identify novel resistance mutations and understanding how metabolic variations influence drug response, will be crucial for advancing CML treatment and improving long-term patient outcomes.

## CRediT authorship contribution statement

**Meriem Lahmouad:** Conceptualization, Methodology, Software, Data curation, Writing – original draft, Visualization. **Zahrae Rachid:** Investigation. **Rawane Bellemrrabet:** Investigation. **Jihane Zerrouk:** Investigation. **Khan Wen Goh:** Formal analysis, Investigation, Writing – review & editing. **Abdelhakim Bouyahya:** Software, Formal analysis, Writing – review & editing. **Youssef Aboussalah:** Conceptualization, Supervision.

## Declaration of competing interest

Authors declare that there is no conflict of interest.
